# Whole blood response to lipopolysaccharide depends on both physiological and genetic factors in dairy cattle

**DOI:** 10.1186/s13567-026-01732-y

**Published:** 2026-04-04

**Authors:** Jérémy Lesueur, Rachel Lefebvre, Frédéric Launay, Didier Boichard, Gilles Foucras, Fabien Corbiere

**Affiliations:** 1 Univ Toulouse, ENVT, INRAE, IHAP, Toulouse, France; 2 Univ Paris-Saclay, INRAE, AgroParisTech, GABI, Jouy-en-Josas, France; 3 Unité Expérimentale du Pin, UE 326, INRAE, Borculo, Le Pin Au Haras, France

**Keywords:** Dairy cow, whole blood assay, ex vivo stimulation, LPS, cytokines, immunosenescence, udder health, ketosis

## Abstract

Immunity plays a pivotal role in productivity, health, and overall welfare in dairy cattle. This study aimed to investigate the genetic and physiological factors influencing the capacity of dairy cows to mount an immune response, with a focus on cytokine secretion and their relationship with lactation rank, milk production, udder health and ketosis-related traits. An approach combining ex vivo stimulation of whole blood using lipopolysaccharide (LPS) and Luminex^®^ technology-based cytokine assay was employed to assess response profiles in dairy cows from a divergent selection for mastitis resistance. Cytokine concentrations increased in relation to the lactation rank in the absence of stimulation. Cows with higher breeding values for somatic cell count exhibited lower CCL3 chemokine secretion and cytokines related to type 2 (IL-4) and type 3 (IL-17A) cell-mediated responses, suggesting a biased adaptive response against udder infections. Upon LPS stimulation, higher concentrations of β-hydroxybutyrate (BHB), indicative of ketosis, were associated with altered cytokine secretion patterns, especially with increased pro-inflammatory cytokines. In addition, cows with high milk production exhibited reduced basal secretion of inflammatory cytokines and displayed a capacity for a more balanced adaptive immune response under the LPS condition. Clustering analyses confirmed the cumulative effect of lactation rank and occurrence of ketosis on the response patterns. This study provides novel insights into how factors influence the immune response in dairy cows, offering potential implications for targeted interventions and breeding strategies to enhance disease resistance and overall animal health in dairy farming practices.

**Supplementary Information:**

The online version contains supplementary material available at 10.1186/s13567-026-01732-y.

## Introduction

Maintaining animal health and welfare while maximising productivity is becoming increasingly important to ensure the profitability and sustainability of the dairy sector. The frequent occurrence of infectious and metabolic diseases [1], which are strongly interrelated, remains an important factor affecting the overall health, welfare and performance of dairy cows. Diseases are partly due to predisposing factors related to herd management and genetic background. There are many interactions between these factors, which makes it difficult to determine which factor controls which disease. Several mechanistic hypotheses have been proposed to explain the increased predisposition of dairy cows to diseases such as metritis, mastitis, lameness, ketosis and abomasal displacement in the first weeks after calving. Some emphasize metabolic factors such as energy deficit and associated ketosis [2] or subclinical hypocalcemia [3] as the main triggers. Others favour systemic inflammation as the main cause of maladaptation to the lactation stage [4]. Both phenomena are probably intrinsically related, and it is almost impossible to separate them. Their prevention remains challenging to implement because the primary causes are not yet fully understood and precise, and the disease pathways are still under debate.

A predisposing factor to infectious disease is the animal’s inability to fight infection and mount an effective and protective immune response, resulting in inflammation-related symptoms upon infection. In infectious diseases such as mastitis and metritis, an early response linked to innate immunity may be sufficient to protect the cow against extracellular bacteria (e.g. *Escherichia coli*, *Staphylococcus* spp., and *Streptococcus* spp., *Trueperella pyogenes*) that are abundant in its environment [5, 6]. Therefore, understanding the factors that contribute to immune responsiveness in cattle is likely key to developing preventive strategies and improving herd health and welfare. Previous studies have shown that immunity in dairy cows is influenced by a complex interplay of factors, particularly in early lactation, but their description remains overlooked. To advance the definition of immunity in dairy cattle, we have adopted a novel strategy using whole blood stimulation with different ligands, such as microbial-associated molecular patterns (MAMPS) and heat-killed bacteria, combined with the measurement of cytokine secretion using a bead-based assay recently developed for ruminants [7]. This methodology provides a powerful tool for investigating secretion patterns in response to different types of blood stimulation with MAMPS. We hypothesised that individual response patterns correlated with traits, which are genetically or physiologically determined. Here, we aimed to assess the relationship between whole blood response to stimulation and factors such as lactation class, milk yield and infectious and metabolic traits.

To achieve this goal, we investigated the response of a group of well-characterised cows with genetic evaluations. Multiple genomic breeding values were available for different traits of interest, in addition to dense phenotyping of production and health traits. These cows were sourced from a divergent selection scheme for mastitis resistance. To capture the cow’s immune response under these relevant conditions, we used lipopolysaccharide (LPS) as a well-defined inducer of the innate immune response. LPS binds to TLR4, one of the pathogen-related receptors of the Toll-like receptor (TLR) family, and its activity has been largely described [8]. LPS stimulates the response of innate cells such as monocytes, which are the main responding cells in the blood. It has been used in cattle and other species, including humans, to induce a systemic inflammatory response.

We provide novel insights into the modulating factors of the LPS response measured in dairy cows. This knowledge helps to identify factors that influence the cow’s ability to respond to infection and may help to delineate the main factors that compromise the immune response at calving.

## Materials and methods

### Study population

The study population consisted of 105 Holstein–Friesian cows from a divergent genetic experiment on mastitis predisposition. Sires used to create the two divergent lines were chosen on the basis of their genomic breeding values for somatic cell count (SCC) and clinical mastitis. They were then divided into two categories: resistant, if the average of the two previous breeding values was greater than 1, and control, if the average was between −1.5 and −0.5; the remaining bulls were not used. The first batch of cows was randomly inseminated with the selected sires. To achieve divergence, their daughters were then inseminated with a sire belonging to the same category as their father.

All cows were housed in a free stall barn at the Pin-au-Haras farm, an experimental dairy cow facility operated by the French National Research Institute for Agriculture, Food and Environment (INRAE). They were fed with a total mixed ration distributed twice a day. Water was available at will. Cows were milked twice a day at approximately 7:30 am and 4:30 pm. Calvings were spread over the year.

Cows in the first (*n* = 42), second (*n* = 43) and third or higher (*n* = 20) lactation will be referred to as L_1_, L_2_, and L_>3_, respectively. Among them, 28 cows were pregnant at the time of sampling, which occurred at an average of 23 ± 18 days of pregnancy. The cows were confirmed to be free from zoonotic infections such as brucellosis, tuberculosis and enzootic bovine leukosis following mandatory herd screening. Their health status was assessed daily throughout the entire lactation period. From 2 weeks before to 2 weeks after blood sampling, six cases of clinical mastitis, one case of cystitis and one case of lameness were reported, and the affected cows were subsequently removed from the study. The cows were managed in a single herd, ensuring that they were exposed to the same environmental factors.

### Experimental design

The experimental design of the study followed a crossover design where each cow served as its own control. The treatment involved two modalities: stimulation with a predefined concentration of LPS (3 µg/mL final) or a null condition with diluent only.

### Data collection

#### Husbandry data and genomic breeding values

The cows were genotyped with the EuroG10k chip (Illumina Inc., San Diego, CA, USA). The genomic estimated breeding value (gEBV) for milk yield (gEBV_MY_), SCC (gEBV_SCC_), body condition score (gEBV_BCS_) and functional longevity (gEBV_FL_) were predicted from the genotyping data [9, 10].

#### Phenotypic data

Phenotypic data used in the study included milk yield, fat and protein content of milk, SCC, milk β-hydroxybutyrate (BHB) concentration and pregnancy status at the time of sampling.

Milk yields were recorded at each milking session while milk composition (fat, protein and BHB) was determined twice a week using mid-infrared spectroscopy. SCC was also measured twice a week with a somatic cell counter (Fossomatic model 90, Foss Food Technology, Hillerod, Denmark).

Pregnancy status was determined by measuring the blood progesterone concentration.

SCC values were normalised through somatic cell scores (SCS) conversion [11]:

$${\mathrm{SCS}}={\mathrm{log}}_{2}\left(\frac{\mathrm{SCC}}{100000}\right)+3$$

To ensure fair comparisons between cows and account for the energy used to produce milk, the fat- and protein-corrected milk (FPCM) was used instead of the raw milk yield. FPCM represents the quantity of standardized milk containing the same amount of energy as the raw milk [12]:

$${\mathrm{FPCM}}={\mathrm{my}}\times \left(0.337+0.116\times {\mathrm{fc}}+0.06\times {\mathrm{pc}}\right)$$

where *my* corresponds to the daily milk yield (kg), *fc* and *pc* correspond to the fat and protein content in percent by weight (%).

To account for the potential effects of the physiological stages on cytokine secretion, FPCM, somatic cell score (SCS), BHB and milk fat-to-protein ratio (FPR) were calculated over several temporal windows:

- FPCM_s_, SCS_s_, BHB_s_ and FPR_s_ corresponding to the nearest values to the blood drawing
- FPCM_305_, SCS_305_, BHB_305_ and FPR_305_ corresponding to the cumulative FPCM, the average SCS, BHB, and FPR over the first 305 days in milk, respectively,
- BHB_0–14_, FPR_0–14_, BHB_50–60_, and FPR_50–60_ corresponding to the average BHB concentrations and FPR over the first 14 days in milk and between the 50th and the 60th days in milk, corresponding to the beginning and peak of the lactation, respectively.

### Sample preparation and stimulation

Blood samples were collected at 101 ± 11 days post-calving to avoid the period of the highest incidence of infectious diseases due to immune depression after calving and stimulated ex vivo as previously described [7]. Samples were collected over eight sessions from March 2017 to January 2018.

### Cytokine and chemokine measurement

The analytes of interest were five cytokines involved in the innate immune response (interleukin-1α (IL-1α), interleukin-1β (IL-1β), interleukin-1RA (IL-1RA), interleukin-6 (IL-6), and tumour necrosis factor-α (TNF-α)), five cytokines of the adaptive response (interleukin-2 (IL-2), interleukin-4 (IL-4), interleukin-10 (IL-10), interferon-gamma (IFN-γ) and interleukin-17A (IL-17A), and four chemokines (chemokine (C–C motif) ligand, CCL2 (MCP1), chemokine (C–C motif) ligand 3 (CCL3) (or MIP1α), chemokine (C–C motif) ligand 4 (CCL4) (or MIP1β) and chemokine (C–X–C motif) ligand 10 (CXCL10) (or IP10)).

Cytokines and chemokines median fluorescence intensities (MFI) were measured using a custom 15-plex bovine cytokine assay from Merck-Millipore (SPRCUS617, Millliplex^®^ xMAP^®^, Merck-Millipore, France) and the MAGPIX^®^ system (Luminex^®^) as previously described. MFI were determined by the xPONENT software (version 4.2.1324.0, Luminex Corp, Austin TX, USA). Cytokines and chemokines MFI were converted to concentrations using dose–response curves modelled by 4 or 5-parameter logistic functions, as appropriate.

### Descriptive statistics

As age influences numerous production and physiological traits, continuous variables included in the linear regressions (described below) were compared between lactation ranks (1, 2, and ≥ 3) using Wilcoxon rank sum tests and categorical variables were compared using Fisher’s exact tests. Multiple comparison corrections with the Benjamini–Hochberg method were applied.

### Statistical analyses

The relationships between cytokine concentrations (dependent variables) and breeding values and phenotypic data (independent variables) were investigated using linear regression models.

A model was fitted separately for each cytokine in each condition (CTL, LPS and the difference of concentration between both conditions, referred below as *delta* values). To avoid redundancy of information, correlations between independent variables were analysed beforehand using the Pearson correlation coefficient. In the case of a high correlation (> 0.7) between two variables, the one minimising the Akaike Information Criterion (AIC) was preferred. Cytokine concentrations were log_10_-transformed to reduce the heteroscedasticity inherent in the immunoassay. Continuous independent variables were centred and scaled to ease the interpretation of their relative effect on the dependent variables.

The initial models included all breeding values (gEBV_MY_, gEBV_SCC_, gEBV_BCS_, and gEBV_FL_), production (FPCM and FPCM_305_) and functional traits (SCS_305_, BHB_14_, BHB_50–60_, BHB_s_), and parity rank as independent variables.

A backward variable selection was performed with the likelihood ratio test as the selection criterion, with a significance level set at 0.05. Finally, to account for multiple comparisons, the *P*-values of coefficient estimates were corrected using the Benjamini–Hochberg method within each situation (i.e. CTL, LPS and *delta* values). The goodness of fit of each model was assessed through standardised residuals and quantile–quantile diagrams.

To investigate the possibility of cows sharing similar cytokine response profiles, a clustering analysis was performed using the hierarchical clustering on principal components (HCPC) method. This analysis aimed to identify distinct clusters of cows based on their cytokine secretion patterns.

The cytokine concentrations were centred and scaled to ensure comparability and reduce the influence of scale differences.

To reduce the dimensionality of the cytokine secretion data, a principal component analysis (PCA) was performed. The first principal components (PC) that explained, together, more than 60% of the total variance were retained for subsequent clustering analysis.

The retained principal component (PC) from the PCA were then used as input variables for the HCPC method. The number of clusters was determined by the higher relative loss of inertia. However, if the suggested partition contained small clusters (fewer than five individuals), the number of clusters was reduced until it met this latter criterion.

The characteristics of the cows comprising the different clusters were compared pairwise using Wilcoxon rank sum tests and Fisher tests for continuous and categorical variables, respectively. Multiple comparison corrections with the Benjamini–Hochberg method were applied.

### Statistical software

All statistical analyses were performed using R (version 4.4.0) [13]. The dose–response curves were fitted using the *drLumi* package (version 0.1.2) [14], an R package dedicated to multiplex immunoassay data analysis. The variable selection was done using the *stepCriterion* function from the *glmtoolbox* R package [15], a set of tools to analyse generalised linear models. The cluster analysis was conducted with *FactoMineR* [16], an R package designed for multivariate exploratory data analysis and data mining.

## Results

### Individual cytokine basic levels change according to the lactation rank

The main characteristics of the study group are presented in Table 1. Most breeding values of L_1_, L_2_, and L_> 3_ cows were not significantly different except that L_1_ cows had a significantly lower breeding value for SCC (*P* < 0.001) and the lowest breeding value for body condition score, with a significant difference with the L_2_ cohort (*P* < 0.05). In agreement with the breeding values for SCC, L_1_ appeared to have the highest SCS_305_ (*P* < 0.01) and SCS_s_ (*P* < 0.001).

**Table 1:** **Mean ± SD values of phenotypic data (overall and by lactation rank) in 105 Prim-Holstein cows**

	Lactation rank			
	1	2	≥ 3	Overall
Number of cows	42	43	20	–
SCS_305_	3.2 ± 1.3^a^	2.3 ± 1.1^b^	2.7 ± 1.2^ab^	2.8 ± 1.2
MY_305_ (L)	8186 ± 1559^a^	8265 ± 1297^a^	9172 ± 847^b^	8406 ± 1382
SCS_s_	2.4 ± 1.5^a^	1.2 ± 1.7^b^	1.2 ± 1.6^b^	1.7 ± 1.7
MY_s_ (L)	30.5 ± 6.7^a^	34.5 ± 5.2^b^	38.2 ± 4.1^c^	33.6 ± 6.3
FPCM_s_ (L)	29.5 ± 6.2^a^	31.3 ± 4.4^a^	32.7 ± 4.5^a^	30.8 ± 5.3
BHB_s_ (mmol.L^−1^)	0.036 ± 0.028^a^	0.026 ± 0.023^ab^	0.018 ± 0.03^b^	0.028 ± 0.027
FPR_s_	1.2 ± 0.2^a^	1 ± 0.1^b^	1 ± 0.2^b^	1.1 ± 0.2
BHB_14_ (mmol.L^−1^)	0.074 ± 0.056^a^	0.07 ± 0.048^a^	0.051 ± 0.039^a^	0.068 ± 0.05
FPR_14_	1.3 ± 0.2^a^	1.2 ± 0.1^a^	1.2 ± 0.1^a^	1.2 ± 0.2
BHB_305_ (mmol.L^−1^)	0.048 ± 0.018^a^	0.056 ± 0.022^a^	0.045 ± 0.025^a^	0.05 ± 0.021
FPR_305_	1.2 ± 0.1^a^	1.2 ± 0.1^a^	1.3 ± 0.1^a^	1.2 ± 0.1
BHB_50-60_ (mmol.L^−1^)	0.028 ± 0.025^a^	0.05 ± 0.034^b^	0.039 ± 0.035^ab^	0.039 ± 0.032
gEBV_MY_	208.2 ± 509.2^a^	281.1 ± 364.8^a^	372.4 ± 452.4^a^	269.3 ± 443.8
gEBV_SCC_	− 0.3 ± 1.1^a^	0.2 ± 1.1^b^	0.6 ± 0.8^b^	0.1 ± 1.1
gEBV_BCS_	− 0.1 ± 0.5^a^	0.4 ± 0.9^b^	0 ± 0.8^ab^	0.1 ± 0.7
gEBV_FL_	0.2 ± 0.7^a^	0.3 ± 0.6^a^	0.6 ± 0.5^a^	0.3 ± 0.6
FPCM_305_ (L)	7678 ± 1400^a^	7544 ± 1123^a^	8117 ± 861^a^	7707 ± 1209

Pairwise comparisons between lactation ranks were performed using a Wilcoxon rank sum with the Benjamini–Hochberg correction for multiple comparisons. Lactation ranks not sharing the same superscript letter are statistically different (adjusted *P* value < 0.05).

L_> 3_ cows had a significantly higher 305 day milk yield than other cows (*P* < 0.01), as milk production generally peaks at this age. However, the lactation ranks did not differ in FPCM_305_ (*P* = 0.21) or FPCM_s_ (*P* = 0.14).

Around the peak of the lactation curve (6–8 weeks after calving), milk BHB concentrations differed significantly between L_1_ and L_2_ cows (*P* < 0.01), the latter having the highest concentrations. This difference was not observed for FPR (*P* = 0.42). Besides, around the date of the whole blood assay (WBA), BHB concentrations were significantly higher for L_1_ than L_> 3_ (*P* < 0.01). Similarly, the FPR values for L_1_ cows were significantly higher than for other cohorts (*P* < 0.001).

**Figure 1:**
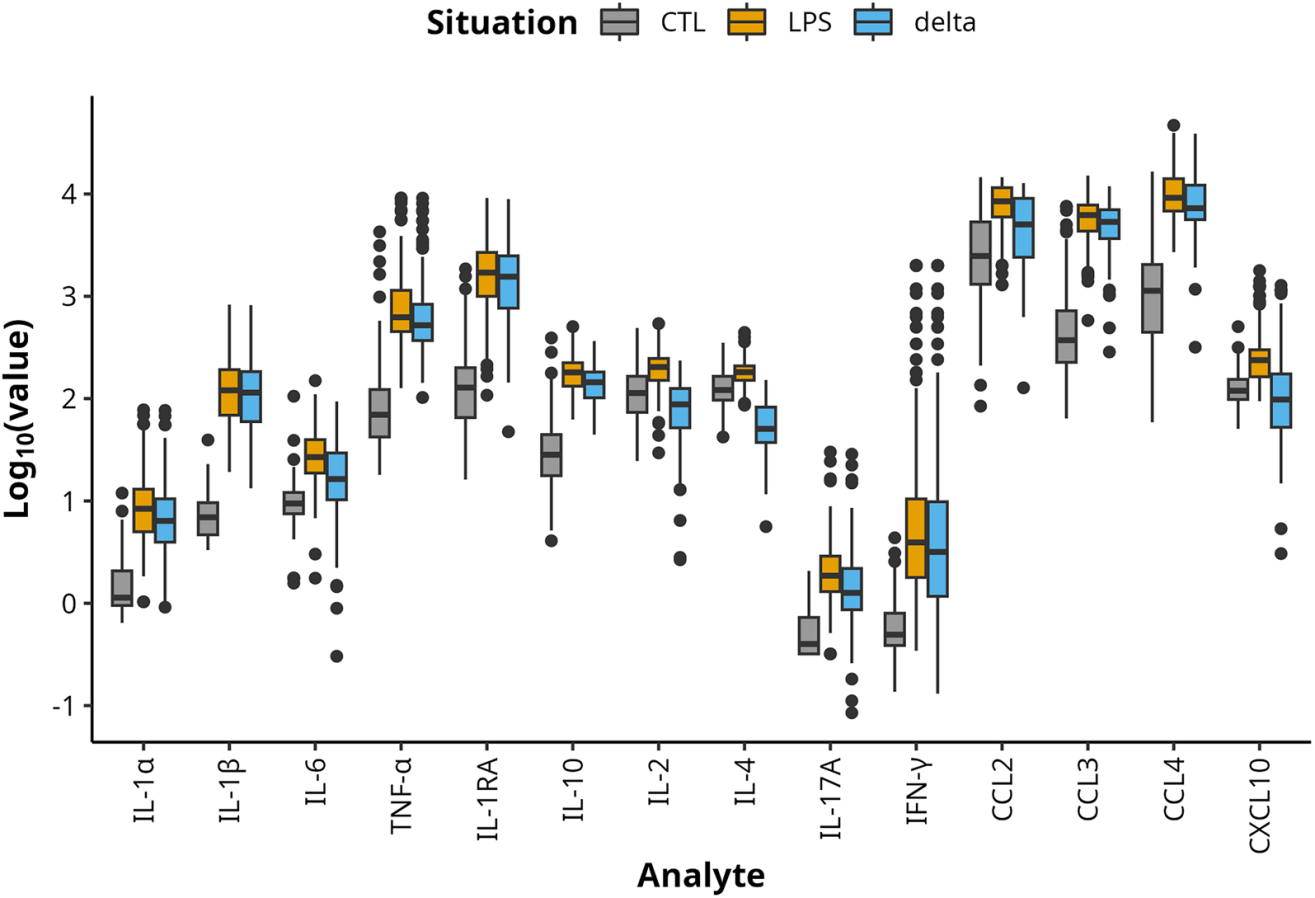
**Boxplot of log10-transformed cytokine concentrations in pg.mL**^**−1**^** without stimulation (CTL) and with LPS stimulation in 105 Prim-Holstein cows**. The delta situation represents the log_10_-transformed concentration differences between both two conditions (LPS–CTL).

As previously shown [5], the LPS stimulation induced cytokine secretions and their concentrations were globally higher than in the absence of stimulation (CTL condition), as shown in Figure 1. Briefly, the CC chemokines had the highest basal and stimulated concentrations, with CCL2 and CCL3 seemingly reaching a ceiling under stimulation. CCL4 had the highest difference between the two conditions (*delta* value). CXCL10 concentrations were low, both in the absence and presence of LPS stimulation. Despite low basal secretions, IFN-γ had the largest secretion range under stimulation while IL-17a had the lowest basal secretion and *delta* values. IL-2 and IL-4 had relatively low *delta* values. Among the cytokines, IL-1RA had the highest concentrations under stimulation and *delta* values, as well as TNF-α, which reached high concentrations after LPS stimulation.

### Lactation rank has a major effect on basal cytokine levels

**Figure 2:**
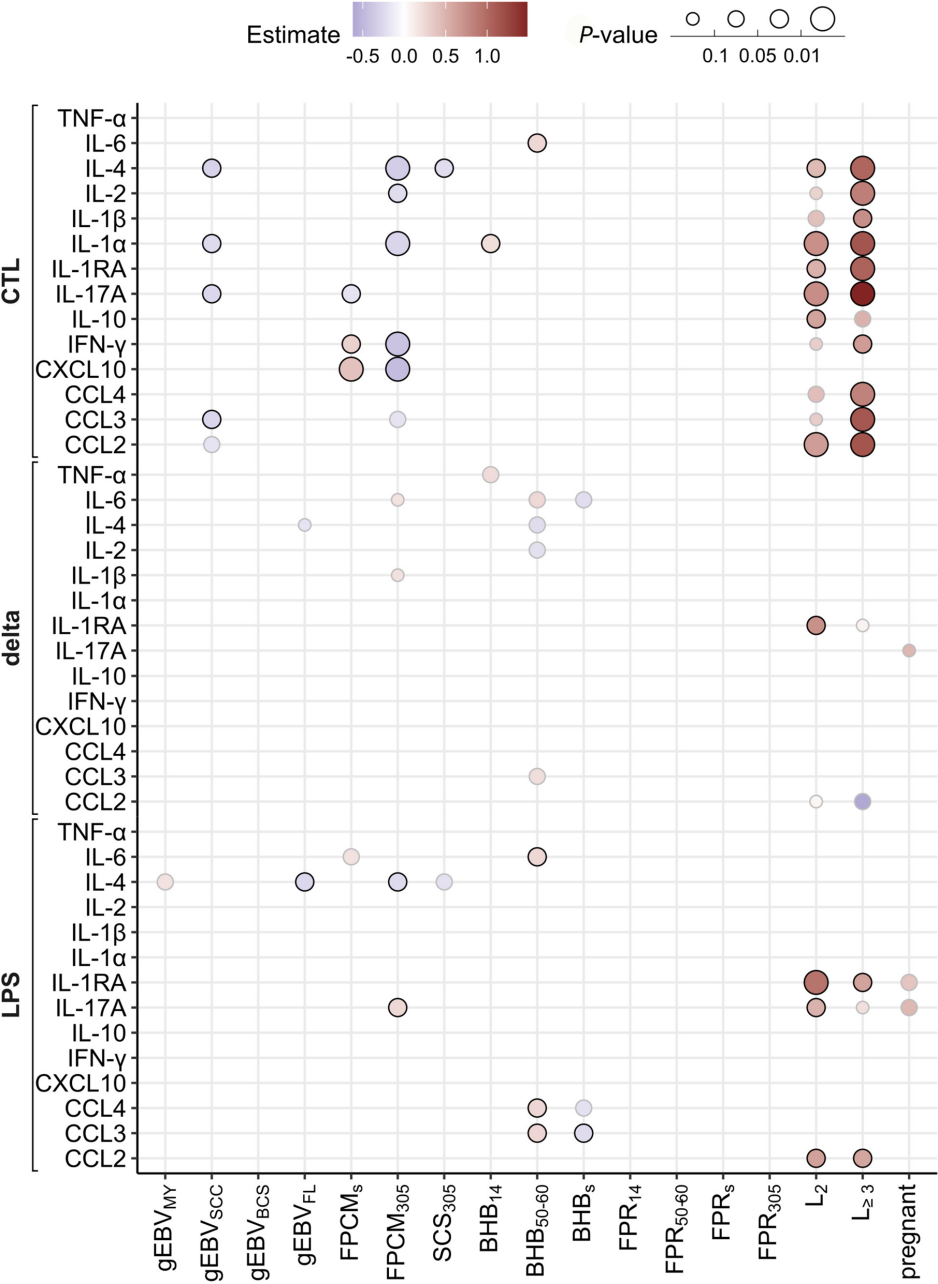
**Summary of linear regressions results after a variable selection with a backward stepwise algorithm and using the likelihood ratio test to retain the best model (*n*** **= 105 Prim-Holstein cows).** *X* and *y*-axis represent respectively the independent variables (production and functional traits) and the analytes for a given situation (CTL, LPS or delta values). Size and colour of a dot correspond respectively to the *P*-value and the estimate of the regression parameter. Independent variables with adjusted *P*-value < 0.05 are circled in black.

The relationships between cytokine concentrations and traits related to milk yield, udder health, ketosis and lactation rank were investigated using linear regression models and results are summarized in Figure 2. In the control (CTL) condition, the lactation rank, the genomic value for SCC (gEBV_SCC_) and the FPCM were significantly associated with the cytokine concentration levels. Overall, the cytokine concentrations increased significantly according to the lactation rank, except for TNF-α, IL-6 and CXCL10. gEBV_SCC_ was negatively associated with IL-4, IL-1α, IL-17a and CCL3. BHB_14_ and BHB_305_ were respectively negatively associated with IL-1α and IL-6 secretions. FPCM_305_ was negatively associated with the secretion of five cytokines: IL-1α, IL-2, IL-4, IFN-γ and CXCL10. Interestingly, FPCM_s_ was positively associated with IFN-γ and CXCL10 but negatively associated with IL-17a.

For the LPS condition, fewer significant relationships between cytokine concentrations and animal traits were found. The lactation rank was positively associated with IL-1RA, IL-17a and CCL2 concentrations. BHB_50–60_ was positively associated with IL-6, CCL4 and CCL3 concentrations, while BHB was negatively associated with CCL3 concentrations. FPCM_305_ was positively and negatively associated with IL-17a and IL-4 concentrations, respectively. gEBV_FL_ was negatively associated with IL-4.

The differences in secretion between the conditions (*delta* values) showed only one significant relationship which was a positive association between lactation rank and IL-1RA concentration.

The previous analyses allowed us to identify redundant relationships between cytokine production and some specific traits, especially the lactation rank, FPCM and gEBV_SCC_ in the absence of stimulation. Altogether these results suggest a high variability of response amongst cows and, potentially, cows sharing the same response profile. This was further investigated by carrying out cluster analyses (HCPC).

### Response profiles are related to the metabolic status and the lactation rank

**Figure 3:**
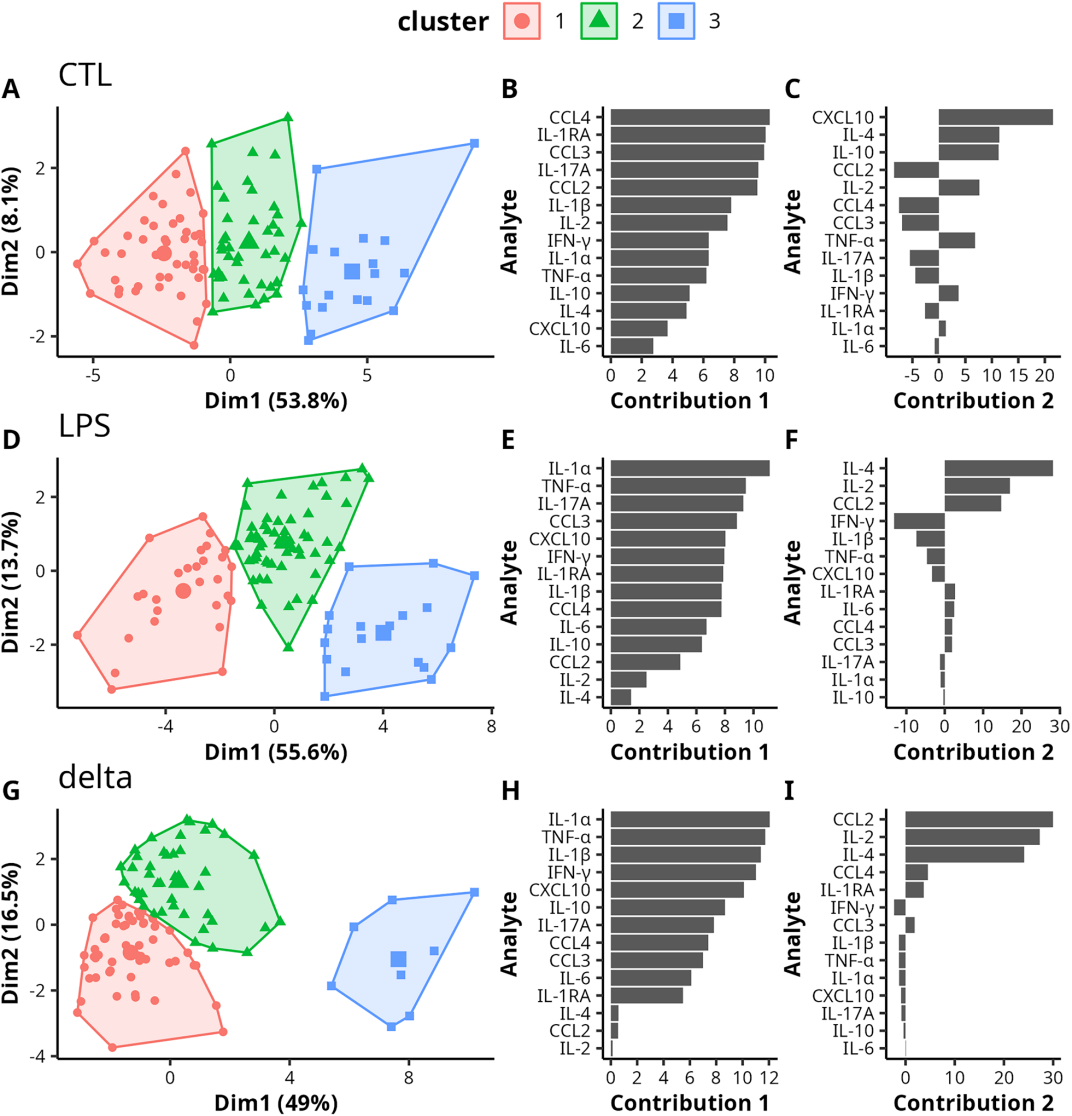
**HCPC on cytokine concentrations obtained for CTL (A–C), LPS (D–F) situations and delta values (G–I) (*n*** **= 105 Prim-Holstein cows).** On the plot of the individuals (**A**, **D** and **G**), each dot represents a cow and its colour and shape correspond to the cluster it belongs to. The *x* and *y* axes correspond to the PCA principal components, with the proportion of variance explained by each principal component shown in brackets. The contribution of the cytokines to the first (**B**, **E** and **H**) and second (**C**, **F** and **I**) principal components is represented by a horizontal barplot, with longer bars indicating a greater contribution of the cytokine to the corresponding component.

For the two conditions (CTL and LPS) and *delta* values, the first two principal components (PC) explained more than 60% of the overall cytokine concentration variance (CTL: 61.9% (Figure 3A), LPS: 69.3% (Figure 3D) and *delta*: 65.5% (Figure 3G)).

In the CTL situation, the cytokines contributing the most to the first PC were CCL4, IL-1RA, CCL3, IL-17a, CCL2, IL-1β and IL-2 (Figure 3B). The cytokines contributing the most to the second PC were CXCL10, IL-4, IL-10, CCL2, IL-2 and CCL4 (Figure 3C). In the LPS condition, the cytokines contributing the most to the first PC were IL-1α, TNF-α, IL-17a, CCL3, CXCL10, IFN-γ, IL-1RA, IL-1β and CCL4 (Figure 3E) while IL-4, IL-2, CCL2, IFN-γ and IL-1β contributed to the second PC (Figure 3F).

For the *delta* values, the cytokines contributing the most to the first PC were IL-1α, TNF-α, IL-1β, IFN-γ, CXCL10, IL-10, IL-17a and CCL4 (Figure 3H). The cytokines contributing the most to the second PC were CCL2, IL-2 and IL-4 (Figure 3I).

In each condition, the hierarchical clustering identified three clusters of unequal size. In the CTL and LPS conditions, the clusters aligned along the first PC, meaning they differed most in the overall level of secretion of almost all cytokines Additional file 1, Additional file 1 and Additional file 3. For *delta*, cluster 3 comprised only eight cows that clearly differed from the other two by higher values on the first component, while cluster 1 (*n* = 55) and 2 (*n* = 42) mostly differed on the second PC (Figure 3G).

Further characterisation of the clusters was performed by comparing production and functional traits using pairwise Wilcoxon rank sum tests and Fisher’s exact tests Additional file 4. The comparisons revealed significant differences in BHB_s_ (0.03 and 0.02 mmol.L^−1^) and BHB_50–60_ (0.03 and 0.05 mmol.L^−1^) concentrations between clusters 1 and 3 for both CTL and LPS conditions, respectively. Additionally, in the CTL condition, cluster 1 contained a higher proportion of L_1_ cows (*n* = 28, 62.2%) than cluster 2 (28.6%, adjusted *P* < 0.01) and cluster 3 (11.1%, adjusted *P* < 0.001), while cluster 3 contained a higher proportion of L_> 3_ cows (*n* = 9, 50.0%) than cluster 1 (6.7%, adjusted *P* < 0.001) and cluster 2 (19.0%, adjusted *P* = 0.04). These findings support the previous results obtained from linear regression models, indicating an increasing gradient of cytokine secretion correlated to the lactation rank in the absence of stimulation. With LPS, cluster 1 was still characterised by a higher proportion of L_1_ cows (60.7%) than cluster 2 (*n* = 16, 27.1%, adjusted *P* < 0.05).

**Figure 4:**
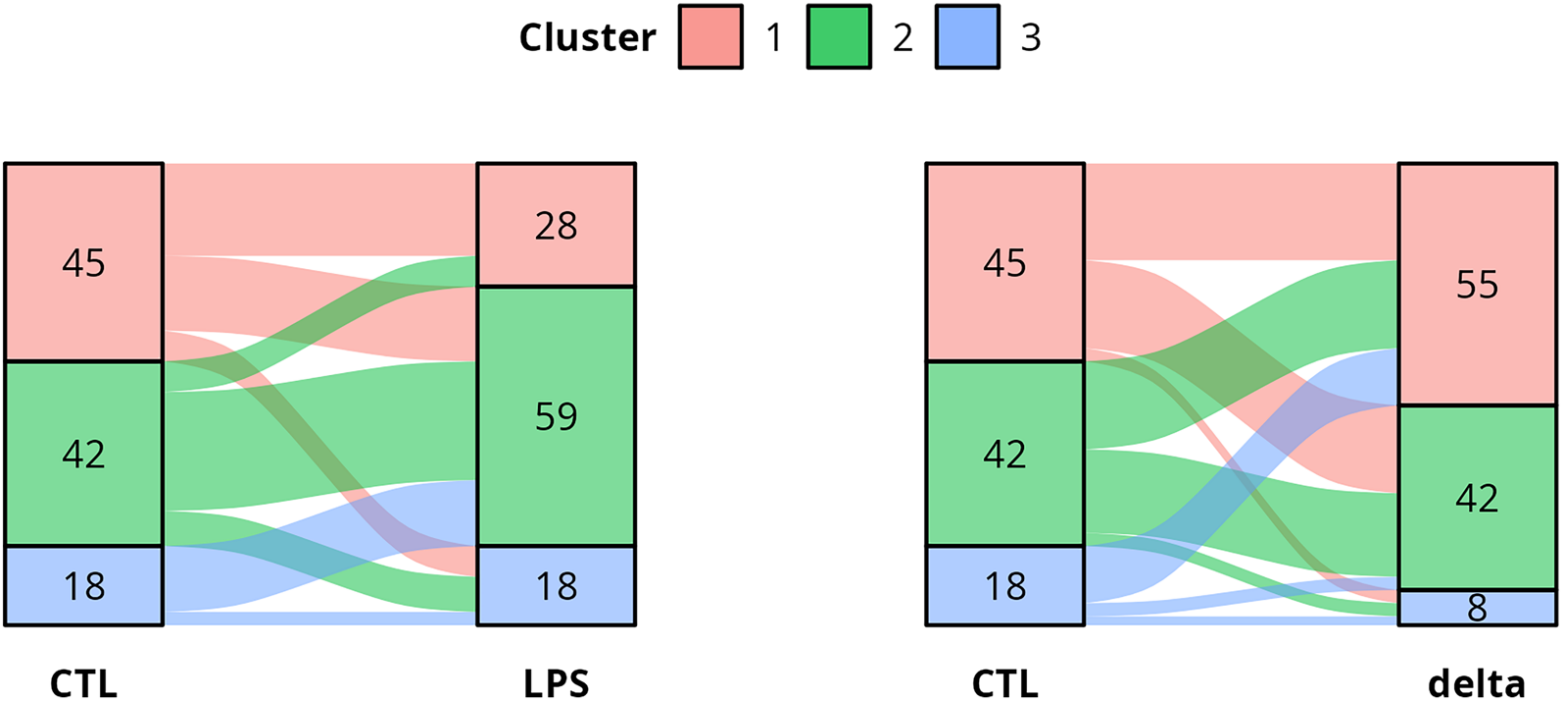
**Tanglegram comparing HCPC clustering between CTL, LPS and** ***delta*** **(*n*** **= 105 Prim-Holstein cows).** For a given line, the thickness is proportional to the percentage of individuals from the left cluster (before stimulation) going to the right cluster (after stimulation). The numbers represent the size of the clusters.

To determine if cows were evenly distributed amongst clusters, we compared cluster composition between conditions and looked for cluster correspondence (described as cow trajectory) (Figure 4). The random nature of cows’ trajectories between their cluster membership in different conditions was assessed using exact multinomial tests. Interestingly, 21 of 28 cows (75%) in the low secretion level cluster under LPS stimulation (cluster LPS 1) also had low basal secretion levels under the CTL situation (cluster CTL 1) (*P* < 0.001). Conversely, only 3 of 18 cows (16.7%) in the high secretion level cluster under CTL situation (cluster CTL 3) were also part of the high secretion level cluster under LPS stimulation (cluster LPS 3) (*P* = 0.01). The fact that most of the cows in the CTL 3 cluster (*n* = 13, 72.2%, *P* = 0.01) were among the cows with the lowest *delta* values (*delta* 1 cluster) also indicates that the cows with the highest basal secretion levels were among the poorest responders to LPS stimulation.

Finally, the LPS cluster 3 (*P* = 0.95) and all *delta* clusters (all *P* > 0.2)were composed of individuals that originated from each of the different CTL clusters indifferently.

## Discussion

The present study aimed to investigate the genetic and environmental factors influencing immune responsiveness to LPS in dairy cattle, with a focus on cytokine secretion patterns and their relationship with lactation rank, milk production and health traits such as mastitis and ketosis. Previous methods developed to assess immune competence relied on adaptative immune response upon immunization [17–20], while innate immunity plays a pivotal role in infectious diseases, especially mammary infections [21, 22]. Dosage of up to three cytokines (IL-1b, IL-6, TNF-α) after whole blood stimulation assay has already been described in cattle to explore innate immunity in cattle [23], but to our knowledge, our study is the first one investigating bovine immune responsiveness using a dosage of a wide panel of 15 cytokines. It allowed us to gain valuable insights into the bovine innate and adaptive immune responses.

Firstly, we observed a positive association between cytokine concentrations and parity in the absence of stimulation, highlighting the complex interplay between lactation and immunity in dairy cows. This relationship is consistent with other studies, which reported an increase in baseline inflammation with parity characterized by higher serum concentrations of TNF-α, IL-6, IL-10, transforming growth factor β (TGF-β) [24], IL-17a [25] and IFN-γ [25, 26] in multiparous compared with primiparous cows. These findings might be related to a phenomenon described in cattle and other species known as “inflammaging” [27–29], a part of a broader phenomenon known as “immunosenescence” [20, 28, 30, 31]. The switch in energetic metabolism along the parity rank is also a strong marker of “inflammaging” [32–34]. Although the underlying mechanism of “inflammaging” is not fully decrypted, it seems to be the result of (i) the persistence of senescent cells, (ii) accumulation of malformed proteins, (iii) leaky gut [35], (iv) a higher number of memory T lymphocytes and a decrease of naive T-helper cells in peripheral blood [36] and/or (v) epigenetic changes [37]. Some of those causes were actually described in Holstein-Friesian cows. For instance, age-related alterations in peripheral leukocyte population, such as a decrease in γδ T-cell activity [26], and a higher B cell [25] but a lower T cell proliferative response [25, 38], were also reported. Concerning age-related epigenetic modifications, some of the changes described in humans are consistent with the increase in cytokine secretions observed in our study. Particularly, age-related loss of methylation of CpG motifs was reported similarly to the TNF promoter of peripheral blood leukocytes [39]. This mechanism is likely to occur in dairy cows and could explain the increase in TNF-α secretion with the parity. Besides, those epigenetic changes are associated with long-term effects on innate immune cell functions, termed “trained immunity” [40, 41], whose effects could be beneficial or detrimental depending on the conditions.

The differential gene expression between older (more than four lactations) and primiparous cows in a transcriptomics study conducted on hepatic tissues from Holstein-Friesian cows revealed an impaired immune response in the older cows [42]. In particular, genes involved in antimicrobial peptides and LPS binding protein productions were upregulated in older cows, suggesting they are more frequently challenged by pathogens, possibly owing to latent or chronic diseases [43, 44], or a lower immunocompetence when facing infection.

Furthermore, our study revealed an association between breeding values for SCC and cytokine secretions. Cows with higher breeding values for SCC exhibited lower secretions of CCL3 chemokine and cytokines involved in type 2 and 3 cell-mediated responses. CCL3 is known to exert an attractive effect on neutrophils [45], macrophages [46] and various T-lymphocytes [47], with these cells being the main contributors to the SCC [48]. Our findings therefore support a broader and more in-depth investigation of the relationship between blood CCL3 concentrations and SCC in milk. Th2 and Th17 cell-mediated responses are components of the adaptive immune response. This could mean that cows with high breeding values for SCC manage to develop an efficient innate immune response to tackle udder infections. Moreover, in our study, cytokine assessments were carried out on peripheral blood. So, in the absence of stimulation, the cytokine concentrations reflected the systemic inflammatory/immune state of the cows at the time of blood sampling. Hence, a lower adaptive immune response could be explained by the potential capacity of high breeding-value cows to regulate inflammation at the local level. Another possible explanation could be the presence of other protective mechanisms or increased tolerance to pathogens, resulting in reduced immune activation [49, 50].

Cows with higher concentrations of BHB, indicative of some negative energy balance, exhibited altered cytokine secretion patterns. Notably, under LPS stimulation, cows with elevated BHB concentrations during peak milk production (40–60 days in milk) exhibited increased secretion of pro-inflammatory cytokines. This finding suggests a potential long-term effect of negative energy balance on the immune response to Gram-negative bacteria. Transcriptomic analysis of neutrophils in dairy cows suffering from metabolic stress reported downregulation of genes linked to apoptosis while genes involved in cell survival were up [51]. These changes suggest that neutrophils survive longer during a metabolic imbalance and that could explain the lingering effect of negative energy balance on the immune response.

Moreover, our study revealed an association between milk production and cytokine secretion. Cows with high FPCM throughout lactation exhibited reduced secretion of several cytokines in the absence of stimulation, indicating lower basal secretion levels. However, in the presence of LPS, these same cows exhibited higher concentrations of IL-17A, suggesting a capacity to deploy a tailored adaptive immune response to extracellular pathogens. This finding suggests that cows with high milk yield throughout lactation may have a more balanced adaptive immune response.

Interestingly, in the LPS-stimulated condition, cytokines involved in the innate immune and inflammatory responses, and the Th17 response appeared to contribute the most to the PCA axes, suggesting a better capacity of this cow’s group to tackle infection by Gram-negative bacteria, as previously shown [52]. In the specific case of LPS stimulation, elevated cytokine secretions (IL-1β, IL-6 and TNF-α) are associated with a reduced blastogenic response of PBMC and an increased susceptibility to diseases during the transition period [49]. If this observation can be transposed to our sampling period, which is likely, these findings would mean the first PC reflects the cow’s susceptibility to infection.

Our clustering analysis provided further insights into cytokine secretion patterns related to lactation rank and BHB concentrations. Indeed, we observed a gradient of cytokine concentrations increasing with parity which is consistent with the results of the linear model regressions. These findings probably confirm a cumulative effect of lactation on the immune system. In addition, in the LPS-stimulated condition, we observed a gradient of cytokine secretion increasing with BHB concentration at the peak of the lactation curve, corroborating the lingering effect of negative energy balance on the immune response to LPS stimulation.

Despite interesting findings that are in strong agreement with previous studies, several limitations should be taken into consideration for interpretation. First, although we assayed 100 cows, the sample size remains relatively small, and only a single herd was considered. Although the group of dairy cows was part of a divergent selection experiment for mastitis susceptibility, a larger multicentric sample size would provide more statistical power and ensure the robustness of our findings. Such a study would also help identify some other relationships between cytokines and variables that were not considered here due to lack of significance in our experimental design. Moreover, the imbalance in the distribution of cows amongst lactation ranks did not permit to evaluate the interactions with other variables. Besides, breed [53] and environmental factors [54–56] have been shown to influence the immune response and patterns of cytokine response; so caution should be exercised when extrapolating these findings to other breeds or breeding systems.

In our study, we used ex vivo stimulation of whole blood samples with LPS to assess cytokine responses. While this approach allows for controlled experimental conditions, it may not fully cover the complex relationship between immune and non-immune cells and the dynamics of the in vivo immune response. Furthermore, our study design was cross-sectional while a longitudinal study would provide a more comprehensive understanding of the changes in cytokine secretion throughout lactation and physiological stages, especially the period around calving.

Although the panel of cytokines has been shown to represent the core of the blood response and provided valuable insights, there are numerous other cytokines and immune markers, such as acute phase proteins [57], that could contribute to a more comprehensive understanding of immune regulation in dairy cows. Moreover, LPS is known to specifically activate TLR4 while there are several other TLRs and signalling pathways [58], such as TLR2 [59], that deserve to be investigated using whole bacteria or other TLR-specific ligands as previously shown [7].

Finally, readers should interpret results from our clustering analyses with caution. Although the cluster membership provided insights into patterns of cytokine secretion, it is based on statistical algorithms and may not fully capture the biological complexity and interactions amongst cytokines. By acknowledging these limitations, we can identify areas for future research. Besides, considering the practical implications of the findings, future studies could explore the potential application of immune competence assessment through cytokine detection in breeding programs and management practices to improve disease resistance and overall animal health. For this purpose, a Genome-Wide Association Study [60] should help to identify the genetic determinants influencing the cytokine patterns observed in our study and to gain more insights into the modulation of the TLR4 signalling pathway.

## Conclusions

This study sheds new light on the environmental and genetic factors that influence immune responsiveness in dairy cows, focusing on cytokine secretion following ex vivo lipopolysaccharide (LPS) stimulation. Our findings suggest that lactation rank has a significant impact on basal cytokine levels, that could be related to the concept of immunosenescence. Metabolic status, particularly negative energy balance, was also found to be linked to altered immune responses, including a more pronounced inflammatory reaction during LPS stimulation. Furthermore, cows with favourable breeding values for somatic cell count exhibited reduced basal secretion of certain cytokines and chemokines, indicating potential differences in baseline immune regulation. These findings pave the way for the integration of immune competence indicators into selection and management strategies aimed at enhancing the health, welfare and productivity of dairy cattle.

## Supplementary Information

**Additional file 1:** **Distribution of cytokine concentrations for each cluster in the absence of stimulation. **Description of data: Boxplot of log10-transformed cytokine concentrations in pg.mL^-1^ for each cluster in the absence of stimulation (*n* =105 Prim-Holstein cows).

**Additional file 2:** **Distribution of cytokine concentrations for each cluster in the LPS situation.** Description of data: Boxplot of log10-transformed cytokine concentrations in pg.mL^-1^ for each cluster in the LPS situation (*n* =105 Prim-Holstein cows).

**Additional file 3:** **Delta values for each cluster. **Description of data: Boxplot of delta values for each cluster (*n* =105 Prim-Holstein cows).

**Additional file 4:** **Distribution of biological parameters at the sampling date and at the peak milk yield**. Description of data: Boxplots of estimated breeding values for milk yield (INLAIT), somatic cell score (INCELL), body condition score (INECPH), β-hydroxybutyrate concentrations in milk at the sampling date, at the peak milk yield, and fat- and protein-corrected milk on the 305 days in milk (*n* = 105 Prim-Holstein cows).

## Data Availability

The datasets used and/or analysed during the current study are available from the corresponding author on reasonable request.

## Abbreviations

BHB: β-Hydroxybutyrate
CCL2: chemokine (C–C motif) ligand 2
CCL3: chemokine (C–C motif) ligand 3
CCL4: chemokine (C–C motif) ligand 4
CTL: control
CXCL10: chemokine (C–X–C motif) ligand 10
FPCM: fat- and protein-corrected milk
IL-10: interleukin-10
IL-17a: interleukin-17a
IL-1RA: interleukin-1RA
IL-1α: interleukin-1α
IL-1β: interleukin-1β
IL-2: interleukin-2
IL-4: interleukin-4
IL-6: interleukin-6
LPS: lipopolysaccharide
MAMPS: microbial-associated molecular patterns
PC: principal component
PCA: principal component analysis
SCC: somatic cell count
SCS: somatic cell score
TGF-β: transforming growth factor β
TLR: toll-like receptor
TNF-α: tumor necrosis factor α
gEBV: genomic estimated breeding value
