# Supplementary material for: Whole blood response to lipopolysaccharide depends on both physiological and genetic factors in dairy cattle
Source: Vet Res. 2026 Apr 4;57:68. doi: 10.1186/s13567-026-01732-y (PMC13154554; doi:10.1186/s13567-026-01732-y)
Supplement: Supplementary file 3 — Additional file 3: Delta values for each cluster. Description of data: Boxplot of delta values for each cluster (n =105 Prim-Holstein cows). [file 13567_2026_1732_MOESM3_ESM.pdf]

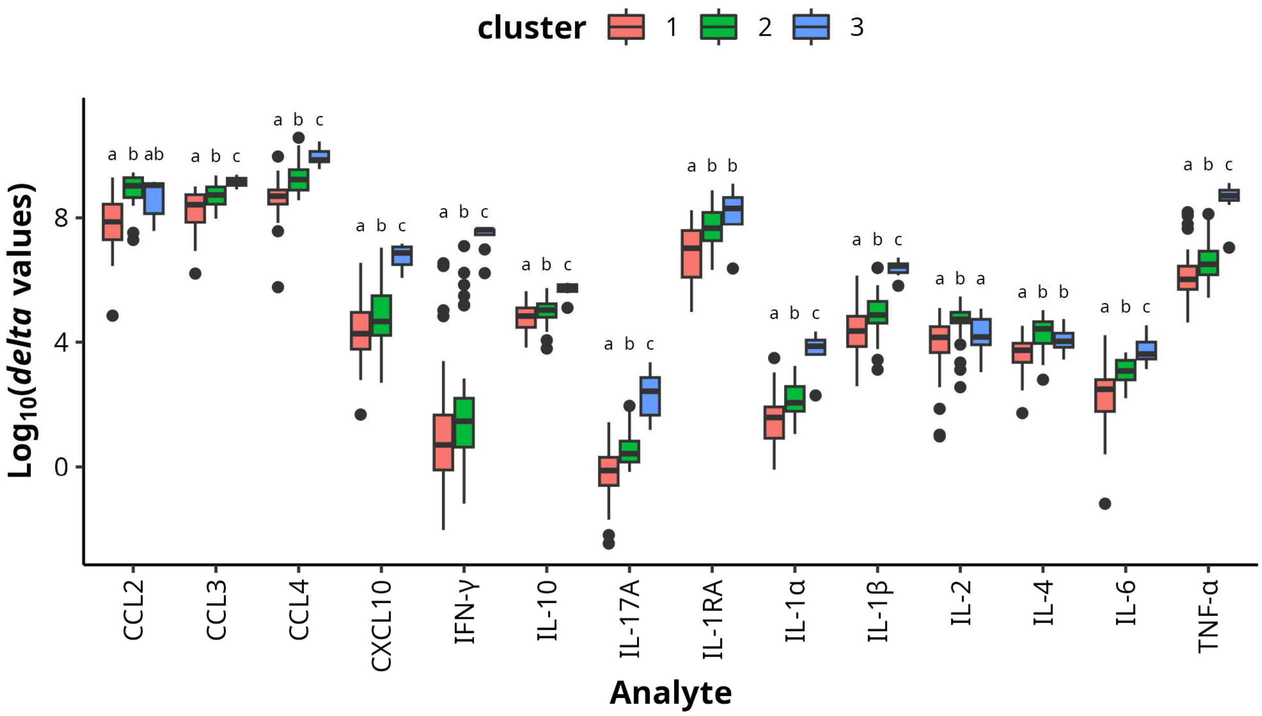

Boxplot of delta values for each cluster (n =105 Prim-Holstein cows). For each cytokine, pairwise comparisons between clusters were performed using a Wilcoxon rank sum test corrected by the Benjamini-Hochberg method. Clusters not sharing the same superscript letter are significantly different.
