# Supplementary material for: Whole blood response to lipopolysaccharide depends on both physiological and genetic factors in dairy cattle
Source: Vet Res. 2026 Apr 4;57:68. doi: 10.1186/s13567-026-01732-y (PMC13154554; doi:10.1186/s13567-026-01732-y)
Supplement: Supplementary file 4 — Additional file 4: Distribution of biological parameters at the sampling date and at the peak milk yield. Description of data: Boxplots of estimated breeding values for milk yield (INLAIT), somatic cell score (INCELL), body condition score (INECPH), β-hydroxybutyrate concentrations in milk at the sampling date, at the peak milk yield, and fat- and protein-corrected milk on the 305 days in milk (n = 105 Prim-Holstein cows). [file 13567_2026_1732_MOESM4_ESM.pdf]

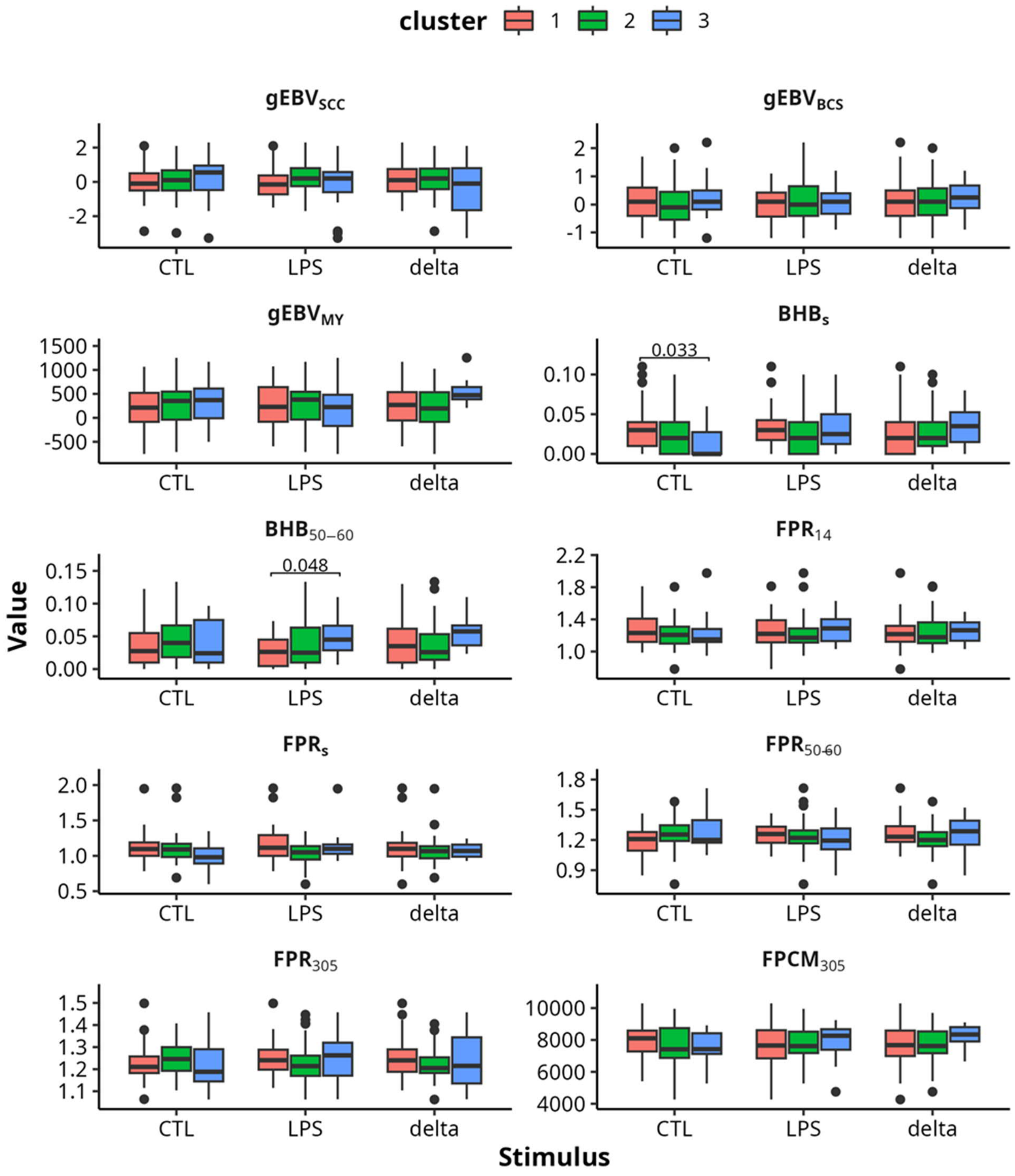

Boxplots of estimated breeding values for milk yield (INLAIT), somatic cell score (INCELL), body condition score (INECPH), β-hydroxybutyrate concentrations in milk at the sampling date, at the peak milk yield, and fat- and protein-corrected milk on the 305 days in milk (n =105 Prim-Holstein cows). The box colors correspond to the cluster determined by the HCPC for CTL, LPS and delta situations. Pairwise Wilcoxon rank sum tests were performed to compare clusters in each situation. Only significant comparisons are shown in the figure (bracket topped with the corresponding *P*-value). P-values were corrected using the Benjamin-Hochberg method for multiple comparisons.
